# Phosphoproteomic comparison of *Pik3ca* and *Pten* signalling identifies the nucleotidase NT5C as a novel AKT substrate

**DOI:** 10.1038/srep39985

**Published:** 2017-01-06

**Authors:** Larissa S. Moniz, Silvia Surinova, Essam Ghazaly, Lorena Gonzalez Velasco, Syed Haider, Juan Carlos Rodríguez-Prados, Inma M. Berenjeno, Claude Chelala, Bart Vanhaesebroeck

**Affiliations:** 1 UCL Cancer Institute, Paul O’Gorman Building, University College London, 72 Huntley Street London WC1E 6DD, UK; 2 Barts Cancer Institute, Queen Mary University of London, Charterhouse Square, London EC1M 6BQ, UK

## Abstract

To identify novel effectors and processes regulated by PI3K pathway activation, we performed an unbiased phosphoproteomic screen comparing two common events of PI3K deregulation in cancer: oncogenic *Pik3ca* mutation (*Pik3ca*^H1047R^) and deletion of *Pten*. Using mouse embryonic fibroblast (MEF) models that generate inducible, low-level pathway activation as observed in cancer, we quantified 7566 unique phosphopeptides from 3279 proteins. A number of proteins were found to be differentially-regulated by *Pik3ca*^H1047R^ and *Pten* loss, suggesting unique roles for these two events in processes such as vesicular trafficking, DNA damage repair and RNA splicing. We also identified novel PI3K effectors that were commonly-regulated, including putative AKT substrates. Validation of one of these hits, confirmed NT5C (5′,3′-Nucleotidase, Cytosolic) as a novel AKT substrate, with an unexpected role in actin cytoskeleton regulation via an interaction with the ARP2/3 complex. This study has produced a comprehensive data resource and identified a new link between PI3K pathway activation and actin regulation.

PI3K signalling is a major regulator of cell growth and metabolism and is one of the most frequently mutated pathways in cancer^1^,^2^,^3^. Two of the most common events are activating point mutations in the p110α isoform of PI3K (encoded by the *PIK3CA* gene), and loss-of-function of the lipid phosphatase and tumour suppressor PTEN^4^,^5^,^6^. Both of these events lead to elevated levels of the phosphatidylinositol(3,4,5)trisphosphate (PIP_3_) lipid, recruitment of PH domain-containing proteins to the plasma membrane and activation of effector proteins including AKT, mTOR and small GTPases such as Rac and RhoA^7^,^8^,^9^,^10^.

Despite activating the same pathway, mutations in *Pik3ca* and inactivation of PTEN are associated with overlapping but distinct tumour profiles and have different outcomes, both in the clinic and in mouse models of cancer^6^,^11^. Molecularly, *Pik3ca* mutant tumours are often dependent on p110α^12^,^13^ while PTEN mutant tumours frequently require p110β activity^14^,^15^,^16^. There is also accumulating evidence that PTEN has phosphatase-independent functions in the nucleus that regulate genomic stability and may be important in tumourigenesis^17^.

While there are a number of PI3K pathway inhibitors in clinical trials, including p110α, AKT, mTOR and pan-PI3K inhibitors, they have so far shown limited efficacy^1^,^18^,^19^. This may be accounted for by the toxicity associated with complete inhibition of PI3K signalling, the existence of feedback mechanisms and the emergence of alternative pathways that can bypass PI3K pathway inhibition^10^. These obstacles highlight the importance of identifying novel players that may contribute to PI3K-driven cell growth.

To dissect the signalling mechanisms after *Pik3ca* mutation or *Pten* loss, we performed a phosphoproteomic screen using a label-free quantitative mass spectrometry method^20^. Label-free mass spectrometry is an alternative to label-based methods such as iTRAQ and SILAC, and has the benefit of allowing analyses of large numbers of samples as well as more consistent quantification of low abundance peptides^21^,^22^.

We were particularly interested in identifying molecular changes that occur at early time points after mutation and in response to low-level activation as these may most closely mimic physiological signalling events. Indeed, in cancer, *Pi3kca* is activated from the endogenous locus and in the heterozygous state, and in the case of *Pten*, often by homozygous loss. We also aimed to assess the impact of *Pi3kca* activation/*Pten* loss at short time points after induction, in the absence of any other oncogenic mutations as well as in the absence of acute stimulation. This contrasts with other studies that examine the effect of PI3K activation in transformed cell lines, at long time points after mutation (when presumably other mutations have occurred) and/or in the presence of acute stimuli. In order to achieve these aims, we took advantage of two inducible genetic models of PI3K activation: primary mouse embryonic fibroblasts (MEFs) with heterozygous expression of the *Pik3ca*^H1047R^ hot-spot mutation from the endogenous locus or homozygous loss of *Pten*^23^. We generated phosphoproteomic profiles of *Pik3ca*^H1047R/WT^, *Pten*^fl/fl^ and appropriate control MEFs.

Using this system we identified 7566 unique phosphopeptides from 3279 proteins. Analysis of regulated phosphopeptides identified events that were commonly regulated by both *Pik3ca*^H1047R^ and *Pten* loss as well as a number of cellular processes that were differentially-regulated by *Pik3ca*^H1047R^ expression or *Pten* loss. Validation of NT5C, one of the hits that was commonly-regulated by *Pik3ca*^H1047R^ and *Pten* loss, demonstrated that it is a novel AKT substrate as well identifying an unexpected role for this protein in regulating actin-dependent processes at the leading edge of the cell.

## Results

### Phosphoproteomic screen of oncogenic PI3K signalling

**Figure 1:**
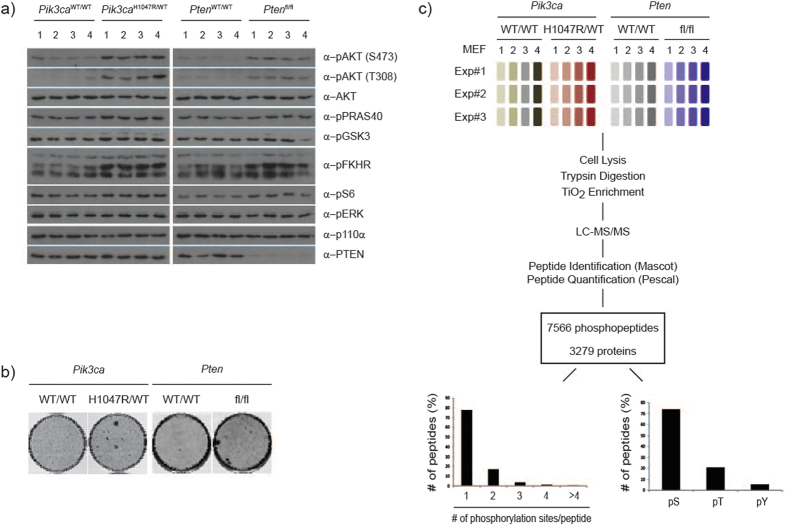
Cellular models and phosphoproteomic workflow. (**a**) Western blot analysis of primary MEF lysates from a representative mass spectrometry experiment. Each lane represents an independent MEF line. (**b**) Focus formation assay of p53-immortalized MEFs of the indicated genotype. (**c**) Experimental design and workflow. Four individual MEF lines were used per genotype and the experiment was repeated 3 times.

In order to map early molecular changes occurring upon PI3K pathway activation, we performed a phosphoproteomic screen using a label-free quantitative mass spectrometry method^20^ on two 4-hydroxytamoxifen (4-OHT)-inducible primary MEF cell models: (1) heterozygous expression of the *Pik3ca*^H1047R^ hot-spot oncogenic mutation from the endogenous locus; further referred to as *Pik3ca*^H1047R/WT^ cells, with wild-type control cells referred to as *Pik3ca*^WT/WT^ and (2) homozygous loss of *Pten*^23^; further referred to as *Pten*^fl/fl^, with wild-type control cells referred to as *Pten*^WT/WT^ (Fig. 1a, Supplementary Fig. S1a). In all experiments, unless otherwise noted, MEFs were 4-OHT-treated.

The use of these two systems allowed us to compare and contrast the effects of two common modes of PI3K pathway activation which have overlapping but distinct effects both biochemically and clinically. To more faithfully mimic the physiologic effect of these genetic PI3K pathway alterations, we performed the screen under basal growth conditions (as opposed to in response to an acute external stimuli), and at short time points after induction (48 h for the *Pik3ca*^H1047R/WT^ screen and 96 h for the *Pten*^fl/fl^ screen; the later time point for the PTEN screen was chosen to allow degradation of the endogenous PTEN protein). Expression of either *Pik3ca*^H1047R/WT^ or loss of *Pten* resulted in a modest but sustained phosphorylation of downstream effectors including AKT, PRAS40, GSK3α/β and FKHR (Fig. 1a) as well as an enhanced ability of cells to form foci (Fig. 1b, Supplementary Fig. S1b).

Taking advantage of the fact that sample number is not a limiting factor in label-free quantitative mass spectrometry approaches, we used 4 independent MEFs per genotype, and repeated the experiment 3 times (Fig. 1c). We therefore analysed 12 samples per genotype for a total of 48 samples overall. This high level of replication was selected to facilitate reproducible detection of small quantitative changes. Following a TiO_2_ enrichment for phosphopeptides, samples were analysed by LC-MS/MS and peptides were identified and quantified as previously described^18^,^21^. Strong quantitative reproducibility was achieved as measured by Pearson Correlation Coefficient which averaged r = 0.83 for experimental replicates and r = 0.87 for biological replicates (Supplementary Table S1). In total we identified 9928 phosphorylation sites. There were 7566 unique phosphopeptides from 3279 proteins of which 78% were singly and 17% were doubly phosphorylated (Fig. 1c, Supplementary Table S2). Of the identified phosphorylation sites, 74% were on serine, 21% were on threonine and 5% were on tyrosine (Fig. 1c). For further analysis, we modelled the data within MSstats statistical framework^22^. Peptides were included that were quantified in at least 6 out of 12 samples in at least one genotype (6970 peptides), and data were log2 transformed and quantile normalised (Supplementary Fig. S1c, Supplementary Table S3).

### Identification of commonly- and differentially-regulated phosphopeptides

**Figure 2:**
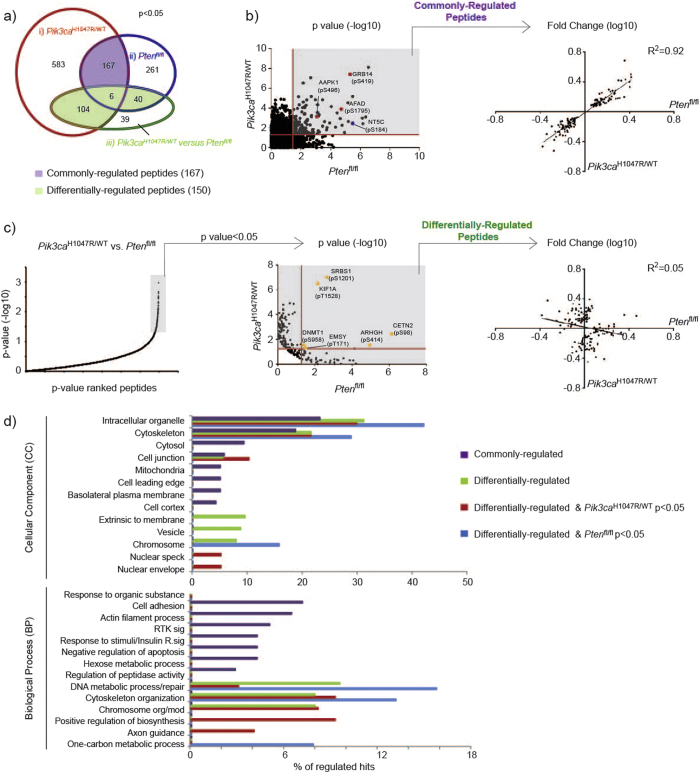
Phosphoproteomic analysis of primary MEFs with inducible PI3K activation. (**a**) Venn diagram of regulated phosphopeptides (p < 0.05) in the three analyses i) *Pik3ca*^H1047R/WT^ (*Pik3ca*^H1047R/WT^ versus *Pik3ca*^WT/WT^); ii) *Pten*^fl/fl^ (*Pten*^fl/fl^ versus *Pten*^WT/WT^) and iii) *Pik3ca*^H1047R/WT^ versus *Pten*^fl/fl^. (**b**) (left) Scatter plot of −log10 transformed p-values from *Pik3ca*^H1047R/WT^ and *Pten*^fl/fl^ analyses. A p-value < 0.05 (red line) was used to define commonly-regulated peptides (shaded in grey). Selected phosphorylation events are highlighted: previously identified PI3K-regulated sites (red dots), validated hit NT5C (blue dot). (right) Changes in phosphorylation intensity from commonly-regulated peptides in *Pik3ca*^H1047R/WT^ and *Pten*^fl/fl^ analyses. Coefficient of determination (R2) is shown. (**c**) (left) Plot of ranked −log10 transformed p-values from *Pik3ca*^H1047R/WT^ versus *Pten*^fl/fl^ analysis. Peptides with p-value < 0.05 are highlighted in grey. (middle) Phosphopeptides with p-value < 0.05 from *Pik3ca*^H1047R/WT^ versus *Pten*^fl/fl^ analysis were re-plotted according to their p-values from *Pik3ca*^H1047R/WT^ and *Pten*^fl/fl^ analyses. Peptides with p-value < 0.05 (red line) in either analyses were defined as differentially-regulated (highlighted in grey). Phosphopeptides that had a p-value < 0.05 in both the *Pik3ca*^H1047R/WT^ and *Pten*^fl/fl^ analyses are highlighted (yellow dots). (right) Changes in phosphorylation intensity from differentially-regulated peptides in the *Pik3ca*^H1047R/WT^ and *Pten*^fl/fl^ analyses. Coefficient of determination (R2) is shown. (**d**) Percentage of regulated hits enriched within cellular component or biological process categories (p < 0.05). Abbreviations used: RTK (Receptor Tyrosine Kinase); sig (signaling); R (Receptor); org/mod (organization/modification).

We defined a phosphopeptide to be regulated if changes in phosphorylation intensity (i.e. increases or decreases in phosphorylation) within a screen, or between the two screens, had a p-value of less than 0.05 (p < 0.05) (Supplementary Table S4). From the resulting analysis, there were i) 860 regulated phosphopeptides in the *Pik3ca*^H1047R/WT^ analysis, ii) 474 regulated phosphopeptides in the *Pten*^fl/fl^ analysis and iii) 189 regulated phosphopeptides in the *Pik3ca*^H1047R/WT^ *versus Pten*^fl/fl^ comparison (Fig. 2a). In all three analyses the fraction of regulated sites with either enhanced or decreased phosphorylation was roughly 50%.

We were particularly interested in identifying phosphosites that had either the same or different patterns of regulation in *Pik3ca*^H1047R/WT^ or *Pten*^fl/fl^ cells, which we defined here as either “commonly-regulated” or “differentially-regulated”, respectively (Fig. 2a). Commonly-regulated peptides were defined as those that had a p-value < 0.05 in *both* the *Pik3ca*^H1047R/WT^ and the *Pten*^fl/fl^ analyses (Fig. 2b). Differentially-regulated peptides were those that had (1) a p-value < 0.05 in *either* the *Pik3ca*^H1047R/WT^ or the *Pten*^fl/fl^ analyses, and (2) a p-value < 0.05 when comparing changes in phosphorylation intensity *between* the *Pik3ca*^H1047R/WT^ and the *Pten*^fl/fl^ screens (Fig. 2c). There were six peptides that were identified as being differentially-regulated as well being regulated in both the *Pik3ca*^H1047R/WT^ and the *Pten*^fl/fl^ screens (i.e. also commonly-regulated) and these were included in the differentially-regulated list.

Using these criteria, we identified 167 commonly-regulated phosphopeptides, including known PI3K-regulated sites such as Grb10 (pS500, pS501, pS503), Afadin (pS1795), and AAPK1 (pS496) (Fig. 2b, Supplementary Table S5). Further analysis indicated that all of the commonly-regulated peptides had changes in phosphorylation intensity that moved in the same direction in the two screens (i.e. enhanced or decreased phosphorylation in both screens). There was also a positive correlation (R^2^ = 0.92) between changes in phosphorylation intensity from the two screens, indicating that these peptides were in fact regulated in similar ways and are strong candidate PI3K effectors (Fig. 2b).

We also identified 150 phosphopeptides that were differentially-regulated in *Pik3ca*^H1047R/WT^ and *Pten*^fl/fl^ cells (Fig. 2c, Supplementary Table S6). In support of these phosphopeptides being regulated independently by *Pik3ca*^H1047R^ expression and loss of *Pten*, we did not observe any correlation (R^2^ = 0.05) between changes in phosphorylation between the two screens (Fig. 2c). The majority of these phosphopeptides were found to be regulated in only one of the screens while there was little to no change in phosphorylation seen in the other screen. There were 6 differentially-regulated phosphopeptides that exhibited phosphorylation changes in both the *Pik3ca*^H1047R/WT^ and *Pten*^fl/fl^ screens. Four of these phosphorylation events (ARHGH (pS414), CETN2 (pS98), KIF1A (pT1528) and SRBS1 (pS1201)) were regulated in the same direction (i.e. upregulated in both screens or down-regulated in both screens), suggesting that they may in fact be commonly-regulated sites, differing only in the amplitude of their phosphorylation or the abundance of the total protein. However, there were two phosphosites, DNMT1 (pS958) and EMSY (pT171) that were regulated in different directions, with increased phosphorylation in *Pik3ca*^H1047R/WT^ cells and decreased phosphorylation in the *Pten*^fl/fl^ screen. These proteins may represent a novel group of effector proteins that are regulated independently by different modes of PI3K activation.

To better understand the biological significance of these results, we used the functional annotation tool DAVID^24^ to identify biological processes that were enriched for proteins that contained regulated phosphorylation events. We compared the results from the commonly-regulated and differentially-regulated lists. Differentially-regulated peptides were analysed as both a single group and as separate categories of *Pik3ca*- or *Pten*-regulated proteins (Fig. 2d, Supplementary Tables S7 and 8). Proteins associated with the cytoskeleton were enriched in all categories, suggesting that this cellular component is very sensitive to alterations in PI3K signalling (Supplementary Table S9). Validating our approach, commonly-regulated proteins were enriched in processes and signalling pathways associated with PI3K activity, including receptor tyrosine kinase and insulin signalling, cellular metabolism and regulation of apoptosis. In addition, we observed enrichment in proteins associated with the mitochondria and the leading edge.

Analysis of differentially-regulated proteins (Fig. 2d) produced strikingly different results. Indeed, there was little overlap in the pathways that were enriched between the commonly and differentially-regulated hits. Processes that may be regulated differently by expression of *Pik3ca*^H1047R/WT^ or loss of *Pten* include those related to DNA organization and metabolism (including histone modification and DNA repair), and interestingly, proteins associated with cytoplasmic vesicles. Further analysis of differentially-regulated hits, highlighted some specificity, whereby *Pik3ca*^H1047R^ expression was associated with regulation of proteins localized to nuclear specks and the nuclear envelope, while *Pten* loss was predominantly associated with proteins involved with DNA metabolism and repair.

We also analysed the regulated phosphosites for overrepresented pSer motifs using the motif-x algorithm^25^,^26^ which analyses the sequences that surround identified phosphorylation sites and identifies overrepresented motifs. Based on our knowledge of kinase consensus sequences we would hope to use the overrepresented motifs to identify key kinases that are regulated by *Pik3ca*^H1047R/WT^ or loss of *Pten*. Commonly-regulated phosphosites were enriched in two motifs, RXXS and SP (Supplementary Fig. S2). RXXS is the consensus substrate motif for AGC kinases including PKA, p70S6K, SGK and AKT^27^. Since AKT preferentially phosphorylates an extended motif (RXRXXS)^28^, this indicates that PI3K activation regulates a number of AGC kinases in addition to AKT. Differentially-regulated phosphosites were also enriched for the SP motif (Supplementary Fig. S2). The relevance of this is unclear. Even though the majority of kinases strongly disfavour proline at position +1, roughly 25% of identified phosphorylation sites in global phosphorylation studies are within SP or TP motifs. This suggests that proline-directed kinases such as GSK, CDK and ERK are more active, or have a larger number of substrates than other kinases^29^.

Finally, we used Scansite^30^ and PhosphoSitePlus^®^ 31 to identify putative or known kinases for the regulated phosphorylation sites (Supplementary Tables S4 and 5). This analysis highlighted the broad effect that PI3K signalling has in cells, as we identified phosphorylation events that are predicted to be modulated by a number of different kinases including AKT, AMPK, CAMK2G, CDK5 GSK3 and PKA. Interestingly, we identified three differentially-regulated phosphorylation sites that either conform to the AKT consensus site (EMSY (pT171), ZNRF2 (pS75)) or is a known AKT site (NIBAN (pS601))^32^. In addition, we also found four previously unidentified AKT consensus sites on FA53B (pS168), K0284 (pS1166), NT5C (pS184) and PKHG3 (pS1134) which were commonly-regulated phosphorylation events.

### Validation of NT5C as a novel AKT substrate

**Figure 3:**
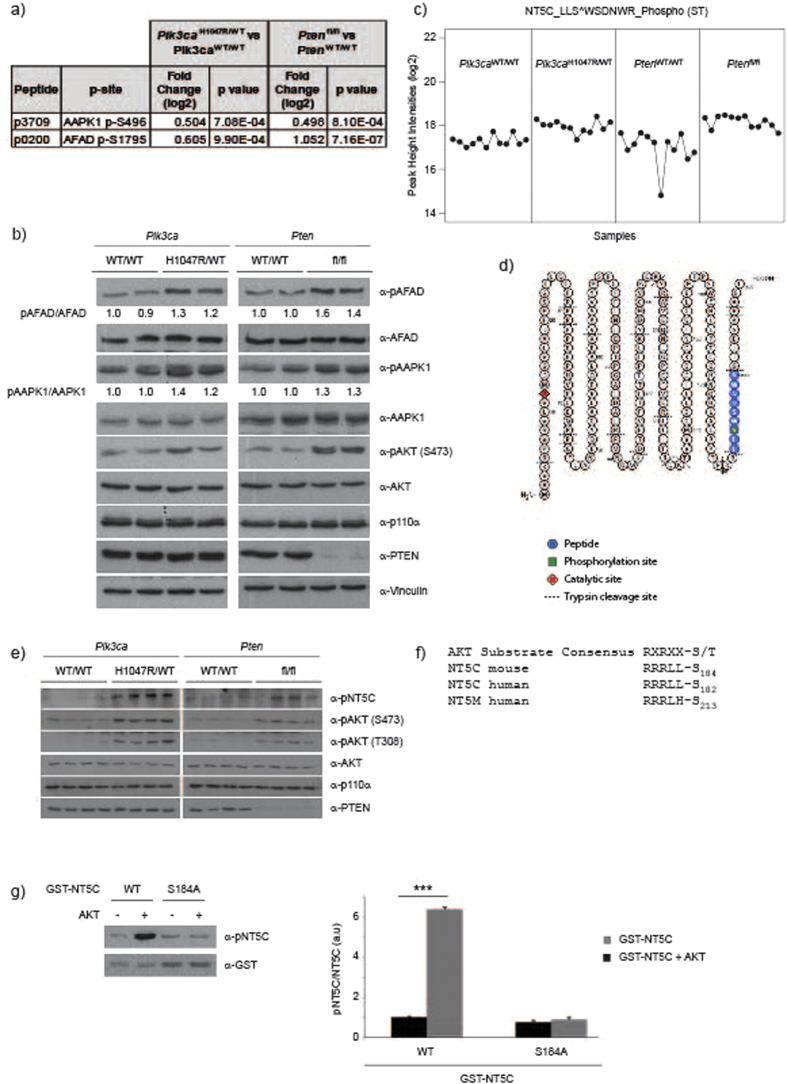
Validation of NT5C as a novel AKT substrate. (**a**) Fold change and p values for indicated phosphorylation sites. (**b**) Western blot validation of pS1795 AFAD and pS496 AAPK1. Ratio between phospho (p) and total protein is indicated. (**c**) Normalized log2 peak height intensities for NT5C peptide#5117 (p5117), LLS^WSDNWR, containing S184. (**d**) NT5C amino acid sequence depicting trypsinization sites and quantified peptide highlighted in blue. Catalytic residue is highlighted in red and S184 phosphorylation site is highlighted in green. (**e**) Western blot analysis of NT5C pS184 from lysates used for mass spectrometry. Western blot panels from Fig. 1a have been reused. (**f**) Alignment of AKT substrate consensus site with NT5C and NT5M. (**g**) AKT *in vitro* kinase activity towards NT5C. Recombinant active AKT (200 ng) was incubated with bacterially produced NT5C (1 μg). Phosphorylation was detected by immunoblotting with indicated antibodies. Quantification of 3 independent experiments, values are expressed relative to GST-NT5C without AKT. Error bars are sem, ***p < 0.001.

In order to validate our mass spectrometry screen we first confirmed the results on two phosphorylation sites for which commercially available antibodies were available, pS496 AAPK (peptide#3709) and pS1795 AFAD (peptide#0200). Both of these sites demonstrated enhanced phosphorylation phosphorylation in both *Pten*^fl/fl^ and *Pik3ca*^H1047R/WT^ cells compared to wild-type controls in both mass spectrometry screens and by immunoblotting (Fig. 3a,b).

Based on the potential biological relevance to PI3K-driven cell proliferation, we focused on NT5C (5′,3′- Nucleotidase, Cytosolic) in order to further validate our mass spectrometry screen. NT5C, which demonstrated enhanced S184 phosphorylation in both *Pik3ca*^H1047R/WT^ and *Pten*^fl/fl^ cells (Fig. 3c,d, Supplementary Fig. S3a) is known to dephosphorylate monophosphorylated nucleotides, with an increased affinity for dNMPs^33^. While its’ *in vivo* role is largely uncharacterized, NT5C is predicted to act as a negative regulator of dNTP pools^33^,^34^. Based on this, our original hypothesis was that PI3K activation may increase cellular dNTP levels through phosphorylation-dependent inhibition of NT5C, ultimately supporting enhanced cell division.

We generated a phospho-specific antibody against phospho-S184 (α-pNT5C) (Supplementary Fig. S3b,c) and found elevated S184 phosphorylation in *Pik3ca*^H1047R/WT^ and *Pten*^fl/fl^ cells by western blot (Fig. 3e), consistent with the mass spectrometry results. Enhanced phosphorylation was observed at both short (2 days) and long (15 days) time points after *Pik3ca*^H1047R/WT^ or *Pten*^fl/fl^ induction (Supplementary Fig. S3d,e). S184 is within a predicted AKT kinase consensus sequence, conserved in human and mouse and in NT5C’s closest homologue, the mitochondrial nucleotidase, NT5M (5′,3′- Nucleotidase, Mitochondrial) (Fig. 3f). To formally test whether NT5C is an AKT substrate, we performed *in vitro* kinase assays using recombinant active AKT, and bacterially-produced GST-NT5C or the non-phosphorylatable mutant GST-NT5C S184A as substrate. Immunoblotting of these kinase reactions with α-pNT5C (Fig. 3g) or an antibody to phosphorylated AKT substrates (Supplementary Fig. S3f,g) demonstrated that AKT can phosphorylate NT5C on S184, and that this is the only AKT phosphorylation site on NT5C.

### NT5C pS184 is sensitive to PI3K inhibition

**Figure 4:**
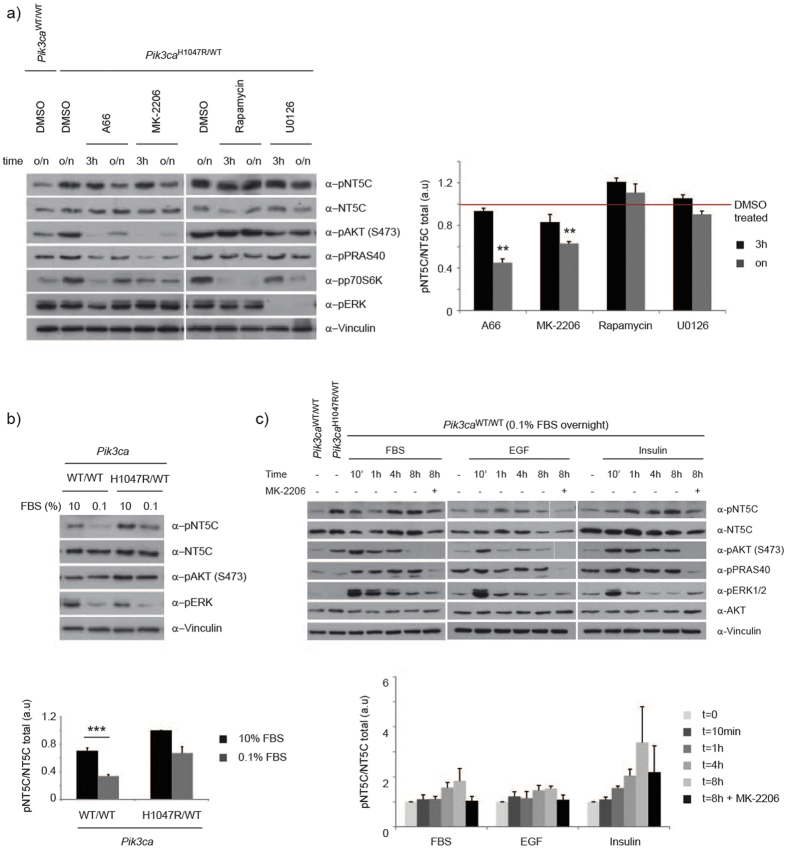
Regulation of NT5C S184 phosphorylation. (**a**) Sensitivity of S184 phosphorylation to PI3K pathway inhibitors. Primary MEFs were treated with A66 (3 μM), MK-2206 (1 μM), Rapamycin (20 nM), U0126 (10 μM) or DMSO as control for 3 h or overnight (o/n) for 16–18 h before lysis. (left) Lysates were immunoblotted as indicated. (right) Quantification of 4 independent experiments. Values are expressed relative to DMSO treated *Pik3ca*^H1047R/WT^ cells. Error bars are sem, **p < 0.01. (**b**) Sensitivity of S184 phosphorylation to growth factor deprivation. Indicated primary MEFs were grown in 10% FBS or starved overnight in 0.1% FBS. (top) Lysates were immunoblotted as indicated. (bottom) Quantification of 5 independent experiments. Values are expressed relative to *Pik3ca*^H1047R/WT^ cells grown in 10% FBS. Error bars are sem, ***p < 0.001. (**c**) Sensitivity of S184 phosphorylation to growth factor stimulation. Primary MEFs were starved overnight in 0.1% FBS and stimulated with FBS (10%), Insulin (100 nM) or EGF (100 ng/ml) for the indicated times before lysis. DMSO or MK-2206 (1 μM) was added at same time as stimuli. (top) Lysates were immunoblotted as indicated. (bottom) Quantification of 3–5 independent experiments. Values are expressed relative to time 0 for each stimuli. Error bars are sem.

We next examined how S184 phosphorylation on NT5C was regulated in cells, by testing its’ sensitivity to various inhibitors and stimuli. S184 phosphorylation was sensitive to inhibition with the PI3K pathway inhibitors A66 (p110α inhibitor) and MK-2206 (AKT inhibitor), but not to rapamycin, U0126 or GSK-650394, which inhibit mTORC1, MEK1/2 and SGK1, respectively (Fig. 4a, Supplementary Fig. S4a). Interestingly, S184 phosphorylation was only sensitive to long-term (overnight) but not short-term (3 h) inhibition, whereas other PI3K-dependent phosphorylation sites, on effectors such as AKT and PRAS40, were inhibited at both time points (Fig. 4a). These results indicate that NT5C phosphorylation is regulated in a different manner than other previously established PI3K effectors such as AKT and PRAS40. For instance, while NT5C phosphorylation was sensitive to growth factor deprivation (Fig. 4b) and was enhanced by stimuli including FBS, EGF and insulin in an AKT-dependent manner (Fig. 4c), its kinetics of phosphorylation were delayed compared to AKT and PRAS40 (Supplementary Fig. S4b). Taken together, our data indicate that NT5C is phosphorylated on S184 in a PI3K-dependent manner, but that this event is not acutely regulated and might require sustained PI3K signalling.

### Phosphorylation on S184 does not regulate NT5C catalytic activity

**Figure 5:**
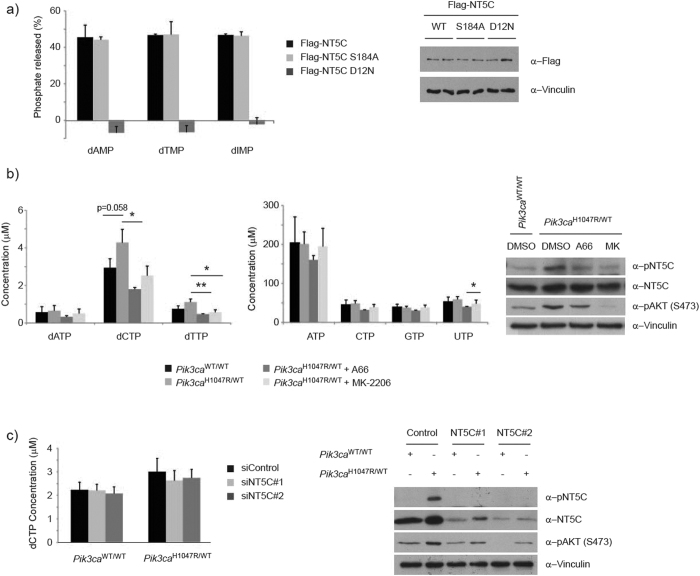
Impact of S184 phosphorylation of NT5C catalytic activity. (**a**) S184 phosphorylation does not regulate NT5C nucleotidase activity *in vitro*. (left) Immunoprecipitates of Flag-NT5C ectopically expressed in HEK293 cells were incubated with 5 mM of the indicated nucleotides. Phosphate release was measured using a malachite green colorimetric assay and expressed as a percent of total nucleotide. The experiment was performed in duplicate and repeated 3 times independently. Error bars are sem. (right) Representative immunoblot from experimental cells. (**b**) Cells expressing *Pik3ca*^H1047R^ have elevated dNTP levels. (left, middle) Nucleotides were extracted from primary MEFs and analysed by UPLC-MS/MS. The experiment was performed in triplicate and repeated 4 times independently. Error bars are sem, *p < 0.05, **p < 0.01. (right) Representative immunoblot from experimental cells. (**c**) Effect of NT5C knockdown on cellular dCTP levels. (left) Nucleotides were extracted from primary MEFs, stably expressing indicated siRNA, and analysed by UPLC-MS/MS. The experiment was performed in triplicate and repeated 3 times independently. Error bars are sem. (right) Representative immunoblot from experimental cells.

We were interested in determining the role of S184 phosphorylation in regulating NT5C function. Based on total levels of protein, S184 phosphorylation did not appear to affect protein stability (Supplementary Fig. S5a). We next examined if S184 phosphorylation regulated enzymatic activity by performing *in vitro* nucleotidase assays, using Flag-tagged NT5C constructs ectopically expressed in HEK293 cells. In line with previous reports^33^, wild-type NT5C, which is strongly phosphorylated on S184 in HEK293 cells (Supplementary Fig. S3f), demonstrated broad substrate specificity against monophosphorylated nucleotides (Supplementary Fig. S5b). NT5C displayed a preference for deoxyribonucleotides over ribonucleotides, with the exception of UMP which was also a strong substrate for NT5C (Supplementary Fig. S5b). However, while a catalytically-inactive mutant (NT5C D12N) was unable to dephosphorylate nucleotides (Fig. 5a), we did not observe any difference in *in vitro* catalytic activity or substrate specificity between wild-type NT5C and the non-phosphorylatable NT5C S184A mutant (Fig. 5a). Therefore, based on these assays, we conclude that S184 phosphorylation is unlikely to regulate NT5C nucleotidase activity *in vivo*.

In order to test the *in vivo* role of NT5C, we used a mass spectrometry-based method to measure cellular concentrations of nucleotides. We first tested the hypothesis that PI3K signalling would increase cellular nucleotide pools by measuring NTP and dNTP levels in primary MEFs expressing *Pik3ca*^H1047R^. We were unable to measure dGTP as it has the same mass and retention time as ATP which is 200 times more abundant than dGTP^35^. We observed a clear trend towards an increase of dCTP, dTTP and UTP, which was sensitive to A66 (p110α inhibitor) and to a lesser degree to MK-2206 (AKT inhibitor) (Fig. 5b). Based on our hypothesis, we predicted that loss of NT5C would mimic PI3K activation and lead to an increase in nucleotide pools. We first explored the impact of NT5C depletion on levels of dCTP for which we had seen the strongest effect of *Pik3ca*^H1047R^ expression. Surprisingly, knockdown of NT5C in either wild-type or *Pik3ca*^H1047R/WT^ cells had no apparent effect on the concentrations of dCTP (Fig. 5c) or other nucleotides tested (Supplementary Fig. S5c). These data suggest that NT5C does not play a major role in regulating nucleotide levels in this cellular system.

### NT5C interacts with the ARP2/3 complex

**Figure 6:**
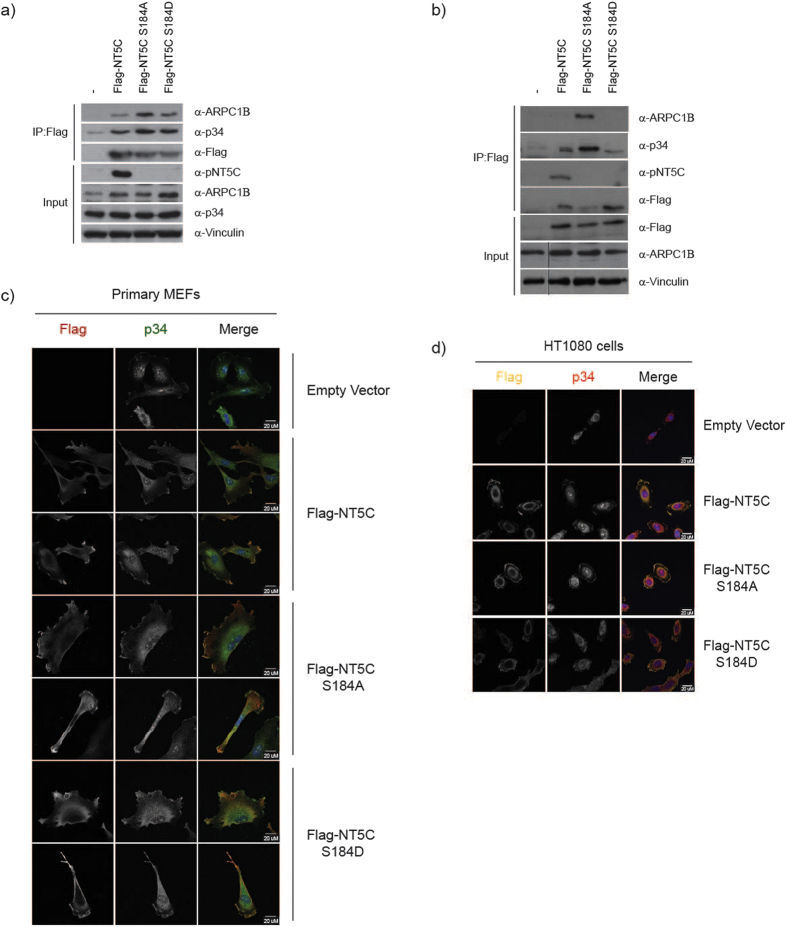
NT5C interacts with the Arp2/3 complex. (**a**) NT5C immunoprecipitates with the ARPC1B and p34 proteins. Flag immunoprecipitates from primary MEFs stably expressing Flag-NT5C and indicated mutants were probed with indicated antibodies. (**b**) NT5C S184A exhibits enhanced binding to ARPC1B and p34. Immunoprecipitates from NIH3T3 cells stably expressing Flag-NT5C and indicated mutants were probed with indicated antibodies. Images cropped from single gel, denoted with black line. (**c**) NT5C localizes to cell edge and colocalizes with p34 in primary MEFs. Cells stably expressing Flag-NT5C constructs were fixed and stained with indicated antibodies. Cells were visualized by confocal microscopy (LSM Zeiss 700). Representative images are shown. (**d**) NT5C localizes to cell edge and colocalizes with p34 in HT1080 cells. Cells stably expressing hNT5C#1 siRNA and Flag-NT5C constructs were fixed and stained with indicated antibodies. Cells were visualized by confocal microscopy (LSM Zeiss 700). Representative images are shown.

We decided to explore whether NT5C might be involved in the regulation of other cellular processes in the context of PI3K signalling. Data from a high-throughput interactome analysis^36^ had identified NT5C as an interacting partner of ARPC1B, a member of the ARP2/3 complex which nucleates branched actin assembly and is important for processes including cell migration and phagocytosis^37^. To test this interaction, we immunoprecipitated Flag-tagged NT5C from cells and probed the immunoprecipitates for ARPC1B. NT5C immunoprecipitates contained ARPC1B (Fig. 6a), validating the results from the interactome screen. In the same immunoprecipitates, we also detected the p34 protein, an integral member of the ARP2/3 complex (Fig. 6a). In the course of our experiments, we frequently observed a strikingly enhanced association between NT5C S184A and p34 or ARPC1B compared to NT5C WT (which is phosphorylated in both MEFs and NIH3T3 cells) or NT5C S184D (a putative phospho-mimetic mutant) (Fig. 6b). While this effect was often stronger in NIH3T3 cells than in MEFs, this indicates that phosphorylation at S184 may regulate the interaction between ARPC1B and NT5C.

We next examined the localization of NT5C in primary MEFs and in human HT1080 fibrosarcoma cells. The latter have large lamellipodia and have previously been used as a model cell system to study the ARP2/3 complex^38^. In both cell types, Flag-NT5C was excluded from the nucleus and exhibited ubiquitous cytoplasmic localization, with prominent staining at the cell edge (Fig. 6c,d, Supplementary Fig. S6a). Moreover, NT5C demonstrated partial colocalization with p34 at the cell edge (Fig. 6c,d) and with the actin-binding protein cortactin (Supplementary Fig. S6a) as well as limited colocalization with actin at the edge of the cell (Supplementary Fig. S6b). Similar patterns of localization were observed with NT5C S184A and NT5C S184D (Fig. 6c,d and Supplementary Fig. S6a,b).

### NT5C regulates cell spreading

**Figure 7:**
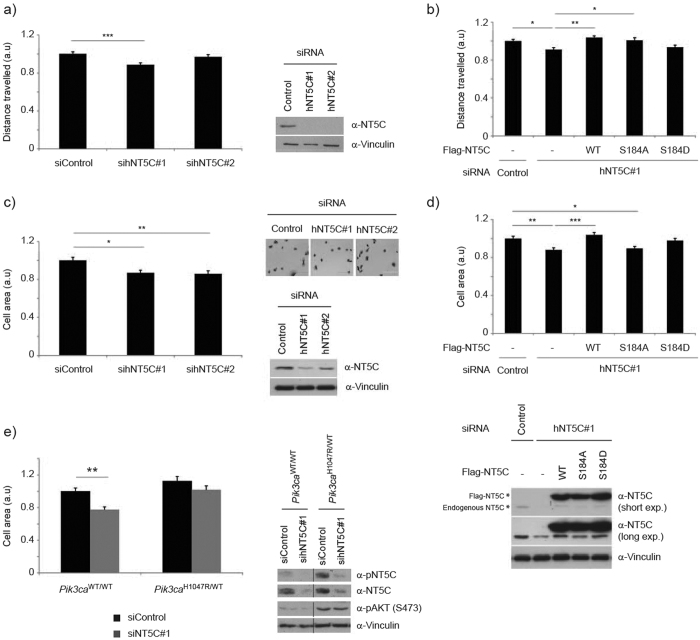
NT5C regulates cell motility and cell spreading. (**a**) NT5C regulates single cell motility. (left) HT1080 cells stably expressing siRNA were seeded into 96-well plates. Images were acquired every 10 min for 80 time points. Distance travelled is expressed relative to siControl cells. The experiment was repeated 3 times independently. n = 235–301 cells per cell line; Error bars are sem; ***p < 0.001. (right) Representative immunoblot from experimental cells. (**b**) NT5C expression rescues motility defect. HT1080 cells stably expressing siRNA and Flag-NT5C rescue constructs were processed as in (**a**). Analysis of distance travelled is expressed relative to cells expressing Control siRNA and empty vector cDNA. The experiment was repeated 4 times independently. n = 138–472 cells per cell line. Error bars are sem; *p < 0.05, **p < 0.01. (**c**) NT5C regulates single cell spreading. HT1080 cells stably expressing siRNA were trypsinized, seeded onto fibronectin coated plates and fixed at 45 min post-seeding. (left) Cell area was determined manually in ImageJ and is expressed relative to siControl cells. The experiment was repeated 3 times independently. n = 326–431 cells per cell line; Error bars are sem; *p < 0.05, **p < 0.01. (top right) Representative image. (bottom right) Representative immunoblot from experimental cells. (**d**) NT5C expression rescues spreading defect. (top) HT1080 cells stably expressing siRNA and Flag-NT5C rescue constructs were processed as in (c). Cell spreading is expressed relative to cells expressing Control siRNA and empty vector cDNA. The experiment was repeated 4 times independently. n = 555–659 cells per cell line; Error bars are sem; *p < 0.05, **p < 0.01, ***p < 0.001. (bottom) Representative immunoblot from experimental cells. (**e**) NT5C knockdown impairs cell spreading in primary MEFs. Primary MEFs stably expressing siRNA were processed as in (**c**) 96 h after 4-OHT treatment. (left) Cell spreading is expressed relative to Pik3caWT/WT cells expressing Control siRNA. The experiment was repeated 3 times independently. n = 244–356 cells per cell line; Error bars are sem; **p < 0.01. (right) Representative immunoblot from experimental cells.

Based on the above data, that NT5C interacts with the ARP2/3 complex and localizes to the cell edge, we explored a possible role for NT5C in regulating ARP2/3-dependent cell processes. We focused on two processes that are linked to ARP2/3 and lamellipodia formation, namely single cell motility and cell spreading^39^. We first tested the effect on single cell motility of stable knockdown of NT5C in HT1080 cells, and found that depletion of NT5C caused a modest but significant decrease in distance travelled per cell (Fig. 7a). This defect could be rescued by re-expression of wild-type NT5C (Fig. 7b). Expression of NT5C S184A led to a partial rescue of the motility defect, whereas NT5C S184D was unable to rescue the defect (Fig. 7b).

We next asked if NT5C was involved in cell spreading, a process that is positively regulated by ARP2/3^39^, by measuring cell area 45 min after cells were plated on fibronectin. Knockdown of NT5C inhibited cell spreading (Fig. 7c) and this was rescued by stable expression of wild-type NT5C (Fig. 7d). Expression of NT5C S184A was unable to rescue the defect while NT5C S184D expression caused a partial rescue (Fig. 7d). Finally, we examined if NT5C could regulate PI3K-dependent cell spreading. Primary MEFs expressing *Pik3ca*^H1047R^ exhibited a slightly enhanced ability to spread following plating on fibronectin, compared to 4-OHT-treated control cells (Fig. 7e). This process was partially inhibited by knockdown of NT5C in *Pik3ca*^H1047R/WT^ cells and more strikingly in *Pik3ca*^WT/WT^ cells (Fig. 7e).

Taken together, our phoshoproteomic screen provides an insight into the complex signalling governed by two common cancer-causing mutations in the PI3K pathway. Our validation of one of these targets has identified NT5C as a novel AKT substrate, and reveals an unexpected role for this protein in ARP2/3-regulated processes such as cell spreading and motility.

## Discussion

PI3K signalling is one of the most frequently activated pathways in cancer. While both *Pik3ca*^H1047R^ expression and loss of function of PTEN enhance PIP_3_ lipid levels and lead to AKT activation, their effect on tumour development both in mouse models and in the clinic displays distinct latency, spectrum and sensitivity to chemotherapy^11^,^40^,^41^. We were interested in understanding the molecular changes caused by these two genetic modes of PI3K pathway activation and, to our knowledge, our study is the first comprehensive phosphoproteomic analysis comparing and contrasting these two prevalent cancer-promoting events.

We used a label-free quantitative mass spectrometry method to obtain unbiased phosphoproteomic profiles from primary MEFs, which either heterozygously express the endogenous *Pik3ca*^H1047R^ mutant allele or have homozygous loss of *Pten*. The aim of our screen was to faithfully mimic mutational activation of the PI3K pathway as observed in cancer. Indeed, in cancer, *Pi3kca* is activated from the endogenous locus and in the heterozygous state, and in the case of Pten, often by homozygous loss. In addition, we attempted to model the effects of *Pi3kca* activation/*Pten* loss at short time points after induction and in the absence of acute stimulation. This contrasts with other studies that examine the effect of PI3K activation in transformed cell lines, at long time points after mutation (when presumably other mutations have occurred) and/or in the presence of acute stimuli. As far as we are aware, such analysis of low level PI3K pathway activation as observed in cancer has not been carried out to date.

Using this system, we identified 7566 unique phosphopeptides from 3279 proteins, with 860 and 474 phosphopeptides regulated in the *Pik3ca*^H1047R^ and *Pten*^fl/fl^ screens, respectively. It is important to note that as samples were collected 48–96 h after mutation induction our screen is not restricted to acute effects of PI3K activation but may also reflect secondary effects which could rely on transcription or translation of regulating proteins or could be caused by changes in target protein abundance. The two categories of phosphorylation events that we were particularly interested in were those that were either commonly- or differentially-regulated between the *Pik3ca*^H1047R^ and *Pten*^fl/fl^ screens. In the category of commonly-regulated phosphopeptides, we identified 167 peptides. These included a number of known PI3K-regulated phosphorylation sites, such as on GRB10, GRB14, Afadin and AMPKα, as well as novel putative substrates of GSK3 and AKT, one of which, NT5C, we characterized in more detail.

We also identified 150 peptides that had markedly different changes in phosphorylation intensity between the two screens. Two of these, DNMT1 (pS958) and EMSY (pT171), were regulated in both genetic contexts, with enhanced phosphorylation in *Pik3ca*^H1047R^ cells and decreased phosphorylation in *Pten*^fl/fl^ cells. The identified site on DNMT1 (DNA (cytosine-5)-methyltransferase 1), pS958, is within a high stringency CDK5 consensus site. Intriguingly, DNMT1 is a known negative regulator of *Pten* transcription and is upregulated in some cancers^42^,^43^. Our mass spectrometric result suggests there might be reciprocal regulation or feedback from PTEN to DNMT1. EMSY (BRCA2-interacting transcriptional repressor EMSY) localizes to sites of DNA repair and acts as a repressor of BRCA2 transactivation^44^. Unexpectedly, not only is T171 within a high stringency AKT consensus site but S173, which is also within the identified peptide, has been previously shown to be phosphorylated by AKT1 and to be important for the interferon response^45^. At present, it is not clear how an AKT substrate could be so differently regulated by *Pik3ca*^H1047R^ compared to *Pten* loss.

Functional annotation of the regulated proteins revealed some further trends. Firstly, we observed that all groups of regulated peptides were enriched for functions related to the cytoskeleton. This is in agreement with a previous phosphoproteomic screen which identified a strong link between oncogenic *Pik3ca* mutation and cytoskeleton rearrangement and cell migration^46^. More specifically, we identified an enrichment of proteins located at the cell cortex and cell edge. This was further supported by our unexpected finding that NT5C also localizes to this cellular compartment.

Our study also identified some processes that may be differentially-regulated downstream of *Pik3ca* and *Pten*. Firstly, there was enrichment of proteins associated with the nuclear envelope (i.e. LMNB1, NU214) and nuclear specks (i.e. PRPF3, SRRM1) specifically in response to *Pik3ca*^H1047R^ activation, indicative of a specific role for p110α signalling in nuclear transport and mRNA splicing. In support of a putative p110α-specific effect in the nucleus, there is evidence that a nuclear pool of PIP_3_ exists, that is associated with nuclear specks and is insensitive to PTEN^47^. Secondly, PTEN loss was associated with enrichment in proteins associated with DNA modification, organization and repair, including the centromeric protein INCE and NBN which is mutated in the chromosomal instability syndrome, Nijmegen breakage syndrome^48^. These data are consistent with previously-documented phosphatase-independent roles of PTEN in regulating genome stability^49^ and may identify additional players involved in this process. Finally, we identified vesicular processes, associated with proteins such as SYTL4 and SNTB2, as being divergently regulated by *Pik3ca*^H1047R^ and *Pten* loss. As phosphoinositide conversion plays an integral role in vesicular trafficking, it is tempting to speculate that gain-of-function of a phosphoinositide kinase (p110α) could have different effects on the composition of phosphoinositide lipid species compared to loss-of-function of the lipid phosphatase PTEN. Taken together, the data from our phosphoproteomic analysis supports further study of the role and importance of subcellular pools of phosphoinositides in diverse cellular processes.

In order to validate our mass spectrometric screen, we focused on NT5C (5′,3′- Nucleotidase, Cytosolic). NT5C p184 was a commonly-regulated phosphorylation event, elevated in both *Pik3ca*^H1047R/WT^ and *Pten*^fl/fl^ cells, and is within an AKT substrate consensus site. Using a phospho-specific antibody we confirmed the mass spectrometric result and demonstrated that S184 in NT5C was phosphorylated by AKT *in vitro* and was sensitive to PI3K inhibitors in cells. Therefore we conclude that NT5C is a novel AKT substrate. However we do not discount the possibility that other PI3K-activated AGC kinases, such as SGK3, may also phosphorylate this S184 in a cell- or context-dependent manner. Despite being sensitive to PI3K inhibition, S184 phosphorylation displayed strikingly different kinetics than other known PI3K effectors such as AKT or PRAS40, in that phosphorylation and dephosphorylation did not appear to be acutely regulated, but instead occurred only after long-term stimulation or inhibition, respectively. Based on this, we speculate that S184 phosphorylation may not be subject to a constitutively active phosphatase and/or may require additional post-translational modifications to occur.

Contrary to our original hypothesis, S184 phosphorylation did not have an impact on NT5C catalytic activity. Even more surprising, while we observed elevated pools of dNTPs in *Pik3ca*^H1047R^-expressing cells, they were not sensitive to NT5C depletion. A caveat with these results is that we were unable to achieve complete knockdown of NT5C by siRNA and the remaining protein may be sufficient to perform its nucleotidase function. However, although contrary to expectations, we propose that NT5C may not be the major 5′ 3′-nucleotidase activity in the cell. In support of this, data from publically available databases show a frequent amplification of NT5C in cancer, at both the transcript and protein level^50^,^51^. We also observed elevated NT5C levels in transformed cell lines compared to non-transformed cell lines such as NIH3T3 and primary MEFs (data not shown). This expression pattern appears counter-intuitive if NT5C was a major negative regulator of nucleotide synthesis, as NT5C overexpression would presumably decrease dNTP pools in cells which have enhanced proliferation rates and thus require elevated nucleotide levels.

Data from an interaction database^36^ prompted us to explore a role for NT5C in regulating the actin cytoskeleton. Our data indicate that NT5C interacts with the ARP2/3 complex, in a manner that may be dependent on S184 phosphorylation of NT5C. A role for NT5C S184 phosphorylation in mediating an interaction with the ARP2/3 complex, is supported by our cell-based studies, where only wild-type NT5C was able to fully rescue the motility and spreading defects caused by NT5C depletion. These defects were modest, suggesting that NT5C is not essential for these processes, but may instead play a regulatory role by modulating the amplitude of the cell’s motility response. The partial rescue by NT5C S184A and NT5C S184D may be indicative of the variable results we had dissecting the Arp2/3 interaction. Of note, particularly in the cell spreading assay, neither NT5C S184A nor NT5C S184D were able to fully rescue the effect of NT5C knockdown. This could suggest the need for dynamic regulation of the phosphorylation site that occurs in a location-specific manner. Based on these results we hypothesize that the NT5C-Arp2/3 interaction regulates Arp2/3 complex activity at the cell edge and that this interaction modulates processes such as lamellipodia formation, cell spreading and cell motility. How exactly NT5C may effect Arp2/3 activity and actin dynamics remains a question for future study.

Taken together, we have produced an unbiased resource of two models of PI3K-regulated phosphorylation. Validation of one of the hits from the screen has identified NT5C as a novel AKT substrate, and point towards an underexplored role for PI3K in regulating actin dynamics at the cell edge. Future work will be important to more fully dissect the interaction between NT5C and the Arp2/3 complex and its role in PI3K-regulated cell motility. Our study adds to the growing realization that PI3K pathway activation in cells leads to an extensive remodelling of the transcriptome and proteome^46^,^52^,^53^,^54^, a phenotype referred to as a ‘butterfly effect’^53^,^55^.

## Methods

### Mutant Mice and Mouse Embryonic Fibroblasts (MEFs) Derived Thereof

Mice were housed in individually-ventilated cages and cared for in accordance with UK Home Office guidelines and legislation, with procedures approved by the Ethics Committees of University College London and Queen Mary University of London. *Pik3ca*^H1047R+neo/WT^ is a mouse line in which one of the two wild-type (WT) *Pik3ca* alleles is converted to *Pik3ca*^H1047R^. Due to the presence of a neomycin (*Neo*) selection cassette in the targeted *Pik3ca* locus, the expression of the mutant p110α^H1047R^ protein is dampened, resulting in minimal or no activation of the PI3K pathway. *Pik3ca*^H1047R+neo^ mice were crossed with *CAG::Flpe-ER*^T2^ mice^56^ that express a germline Flp recombinase that is inducible by 4-OHT. *Pik3ca*^H1047R/WT^; FlpER(T2)^+/−^ mice are further referred to as *Pik3ca*^H1047R/WT^ mice. 4-OHT treatment resulted in the removal of *Neo* cassette, restoration of p110α^H1047R^ expression levels to those of endogenous p110α^WT^ and PI3K pathway activation. The presence of the *Pik3ca*^H1047R+neo^ mutant allele was monitored by PCR using *neo* cassette primers: 5′-GGTTCCAGCCTGAATAAAGCC-3′ and 5′-AGATCAGCAGCCTCTGTTCC-3′, giving rise to an expected PCR fragment of 307 bp. In each amplification cycle, denaturation was performed for 30 sec at 95 °C, annealing for 30 sec at 60 °C, and extension for 1 min at 72 °C; the number of cycles was 35 followed by a final extension of 10 min at 72 °C. Following Flp-mediated recombination the presence of the recombined allele was monitored using recombination primers: 5′-GGTTCCAGCCTGAATAAAGCC-3′ and 5′-CACAGCTGTCCTGGGTAAGG-3′ and the same PCR conditions described above where used. The size of the expected PCR fragments was 425 bp (recombined mutant allele) or 340 bp (wild-type locus). The PTEN^fl^ mouse line in which exon 5 in the phosphatase domain of PTEN is flanked by *loxP* recombination sites, has been described^23^. These mice were crossed onto CreER(T2)^+/−^ mice that express a germline 4-OHT-inducible Cre^57^, generating *Pten*^fl/fl^CreER(T2)^+/−^ mice, further referred to as *Pten*^fl/fl^ mice. MEFs made from E13.5 embryos were treated with 1 μM 4-OHT for one or two consecutive days. Unless mentioned, all experiments with primary MEFs were performed 48–96 h post 4-OHT treatment; control cells were also treated with 4-OHT. Efficiency of recombination was assessed by PCR or western blot analysis.

### Cell Culture

HT1080 cells were cultured in DMEM, high glucose, GlutaMAX™ Supplement (Invitrogen #61965–026) with 10% FBS and penicillin-streptomycin. All other cells were cultured in DMEM with 10% FBS and penicillin-streptomycin. For phosphoproteomic experiments primary MEFs were grown under normoxic conditions. For all other experiments, primary MEFs were grown under hypoxic conditions (5% CO_2_, 3% O_2_) which increases the proliferative lifespan of cultured cells^58^.

### Western blot analysis and antibodies

Whole-cell lysates or immunoprecipitates were resolved by SDS-PAGE and blotted onto polyvinylidene difluoride (PVDF) membranes. Proteins were probed using primary antibodies obtained from the following sources (catalog number between brackets): pAKT (S473) (#9271), pAKT (T308) (#4056), AKT (#9272), pERK1/2 (T202/Y204) (#9106), p110α (#4249), PTEN (#9552), pFoxO1/3a (T24/T32) (#9464), pGSK3 (S21/9) (#9331), pS6 (S240/4) (#2215), pp70S6K (T389) (#9205), pPRAS40 (T246) (#2640), pNDRG1 (T346) (#3217), pAFAD (S1718) (#5485), pAAPK1 (S485) (#2537), AMPK (#2532) and GST (#2624) from Cell Signaling Technology; ARPC1B (#HPA004832), Vinculin (#V9131) and Flag M2 (#F3165) from Sigma; NT5C (#NBP1-84563) from Novus Biologicals; p34 (#07-227) from Millipore and AFAD (ab90809) from Abcam. A rabbit affinity-purified polyclonal antibody against NT5C pS184 (mouse NT5C amino acid numbering, equivalent to human NT5C pS182) was developed in collaboration with GenScript, utilizing the RLLpSWSDNWREILDC peptide, covering amino acids 179–192 of human NT5C. Quantification of western blots was performed using ImageJ.

### Focus Formation Assay

Primary MEFs were immortalized by stable transfection of p53 siRNA^59^. 4-OHT-treated p53-immortalized MEFs were grown to confluence and maintained for 13 days, with regular changes of culture media. Plates were fixed in 3.7% formaldehyde and stained with 0.1% crystal violet followed by extensive washing in water.

### Mass Spectrometry-Based Phosphoproteomics

Samples were processed as described^60^, with the following modifications. Primary MEFs were lysed 48 or 96 h after 4-OHT treatment in 1 ml of urea buffer per 15 cm tissue culture dish. Reduction and alkylation of cysteines was performed at room temperature for 15 min in the dark. For TiO_2_ enrichment of phosphopeptides, packed tips were sequentially washed with glycolic acid buffer 2, 99/1 H_2_O/ACN and eluted with four sequential washes of 5% NH_4_OH in 1% ACN. LC-MS/MS analysis and data processing was performed as described^61^. Briefly, phosphopeptide pellets were resuspended in 20 μl of 0.1% TFA, and 4 μl was loaded into an LC-MS/MS system, which consists of a nanoflow ultrahigh pressure liquid chromatography (UPLC, nanoAccuity, Waters) coupled online to an Orbitrap XL mass spectrometer (Thermo Fisher Scientific). The top five most intense multiply charged ions were selected for collision-induced dissociation fragmentation in multistage activation mode. The resolution of MS1 was set to 60,000.

### Peptide Identification and Quantification

Peptide identification was performed by searching the MS/MS data against the SwissProt database restricted to mouse entries with the Mascot search engine^62^. Mass tolerances were set to 10 parts per million (ppm) and 600 millimass units for parent and fragment ions, respectively. Allowed variable modifications were phosphorylation on Ser, Thr, and Tyr; PyroGlu on N-terminal glutamine; and oxidation of methionine. Phosphopeptides with a Mascot expectancy of < 0.05 (~2% false discovery rate) were included in a database of sites quantifiable by MS. Pescal software^63^ was then used to obtain peak heights of extracted ion chromatograms of phosphorylated peptides in this database across all the samples being compared. Pescal aligned retention times using those of peptides common to all samples as reference points along chromatograms. The extracted ion chromatogram (XIC) windows were 6 ppm and 2 min. Accuracy of phosphorylation site identification is expressed by the max_delta score (Supplementary Table S2).

### Statistical and Functional Analysis of the Phosphoproteomic Data Set

Quantified phosphopeptides were analysed within the model-based statistical framework MSstats v3.3.1^64^. The experimental design was defined by two screens and two conditions per screen. Each condition was replicated three times experimentally and four times biologically. For statistical analysis, peptides were included if they were quantified in at least 6 out of 12 samples in at least one genotype. The data was log2 transformed and quantile normalised. To find differential abundant proteins across conditions, significance analysis consisting of fitting a statistical model and performing model-based comparison of conditions was performed within MSstats. The group comparison function was employed to test for differential abundance between conditions or screens. Unadjusted p-values were used to rank the testing results and to define regulated phosphopeptides within and between screens. The DAVID Bioinformatics Resource (https://david.ncifcrf.gov/summary.jsp) was used to perform functional annotation of regulated peptides, using the complete list of peptides identified within our mass spectrometric screen as background. Categories/Processes were enriched if they had a p-value < 0.05. The motif-x algorithm (http://motif-x.med.harvard.edu) was used to extend and extract motifs^25^,^26^. The significance threshold was set to p-value < 1e−3, minimum occurrence of motif was 20 for pSer peptides using an IPI Mouse proteome background. We used Scansite^30^ (http://scansite.mit.edu/) to identify predicted kinases and PhosphoSitePlus^®^ (http://www.phosphosite.org/)^31^ to identify previously validated kinases for identified phosphorylation sites at high stringency.

### Plasmids, Cloning and Cellular Knock-down of NT5C

NT5C cDNA was amplified from a mouse cDNA library using the following primers [Forward (with added EcoRI restriction site, underlined) 5′-TACGCGAATTCAATGGCGGTGAAGCGGCCCGT-3′; Reverse (with added XbaI restriction site) 5′-ATCCTCTAGATCACAGGCTGGCTCGCTTGC-3′], cloned into the EcoRI and XbaI restriction sites within the multicloning site of 3xFlag-CMV-7.1 and fully sequenced. NT5C was then subcloned into pGEX4T3 and sequenced. Lentiviral plasmids were constructed using the Gateway system as follows: Flag-NT5C was subcloned into the entry vector pEntr1A, sequenced, following by cloning through recombination into the destination vector pLenti CMV Blast Dest (Addgene#17451). Point mutants of NT5C were generated by site-directed mutagenesis and sequenced. For transient knockdown of NT5C in human cells, ON-TARGET plus Human NT5C (30833) siRNA-SMART Pool was purchased from ThermoScientific (L-020511-01-0005). For stable knockdown of NT5C, shRNAmir were purchased from Open Biosystems as follows: non-silencing scrambled (ATCTCGCTTGGGCGAGAGTAAG), shRNAmir against mouse NT5C: NT5C#1 (V2LMM_33051), NT5C#2 (V3LMM_521869), shRNAmir against human NT5C: hNT5C#1 (V2LHS_85392), hNT5C#2 (V2LHS_85394).

### Cell Lysis and Immunoprecipitation

Cells were lysed in CHAPS lysis buffer (40 mM HEPES [pH 7.5], 0.5% CHAPS, 120 mM NaCl, 1 mM EDTA, containing 1 mM DTT, phosphatase (phosSTOP (Roche)) and protease (Sigma) inhibitor cocktails). For analysis of protein interaction, anti-Flag M2-agarose immunoprecipitations were performed overnight at 4 °C. All other immunoprecipitations were performed with anti-Flag M2-agarose or glutathione-Sepharose 4 Fast Flow (GE Healthcare) for 2 h at 4 °C, washed four times with CHAPS lysis buffer and eluted by boiling in sample buffer.

### AKT Kinase Assay

Kinase assays were performed in a 30 μl reaction mixture containing active 250 ng AKT (purchased from Millipore) and bacterially produced GST-tagged NT5C proteins (1 μg) in kinase assay buffer (10 mM MOPS, pH 7.2, 20 mM MgCl_2_, 2.5 mM EGTA, 12.5 mM β-glycerol phosphate, 0.5 mM sodium orthovanadate, 0.5 mM dithiothreitol (DTT)). Kinase assays were initiated by addition of 80 μM ATP and performed for 30 min at 30 °C. Reactions were stopped by addition SDS-PAGE sample buffer, separated by SDS-PAGE and phosphorylation detected by immunoblotting.

### NT5C Enzymatic Assay

Flag-NT5C immunoprecipitates were washed two times in CHAPS lysis buffer and two times with Phosphatase reaction buffer (20 mM MgCl_2_, 50 mM Tris-maleate, pH 6.0, 0.2 mg/ml bovine serum albumin, 5 mM DTT). Phosphatase assays were performed in a 50 μl reaction containing 5 mM of nucleotide substrate in phosphatase reaction buffer. Reactions were carried out for 30 min at 37 °C. Reactions were stopped by addition of 200 μl dH_2_O, centrifuged and placed on ice. Supernatant containing released phosphate was measured using a malachite green-based colorimetric phosphate assay kit (Abcam #ab65622), according to the manufacturer’s instructions. Experiments were performed in duplicate and results averaged.

### Preparation of Lentivirus and Lentiviral Transduction

shRNAmir lentivirus was produced and titrated by the Genomics Facility at UCL and used at a MOI of 10. Lentivirus for NT5C cDNA was packaged in HEK293T cells by FuGENE transfection. Lentiviral plasmid, packaging plasmid (p8.91) and coat protein plasmid (pMDG) were used at a 1.5:1:1 ratio. Viral supernatants were collected after 48 and 72 h, cleared through a 0.45 μm filter and stored at −80 °C. cDNA lentivirus was used at a 1x concentration. For production of stable cell lines, lentiviral transduced cells were selected with puromycin and/or blasticidin.

### dNTP Quantification by UPLC-MS/MS Sample Extraction and Analysis

Intracellular nucleotide pools were quantified according to the previously published method for determination of intracellular gemcitabine triphosphate in human PBMCs^65^. We modified this method and validated it for the measurement of intracellular NTP and dNTPs. Briefly, samples were collected in triplicate and results averaged. Cells were washed in PBS and re-suspended in 100 μl PBS and 100 μl 0.8 M perchloric acid (PCA), vortex mixed and incubated on ice for 30 min. After centrifugation (10,000 *g*, 4 °C for 10 min), 180 μl of supernatant was transferred to a new tube and stored at −80 °C. At the time of analysis, 50 μl of 1 M NH_4_Ac was added to the PCA extract, and the solution neutralised by addition of 20 μl of 10% NH_3_. Finally, 5 μl of internal standard 8-ChloroATP (Sigma) and 5 μl deionised water was added. The extract was transferred to LC-MS vials and 10 μl was injected in to the UPLC-MS/MS system. LC-MS grade water, methanol, acetonitrile and formic acid were from Fisher Scientific, UK. Calibration standards were prepared at 0, 1, 5 and 25 μM.

### dNTP Quantification by UPLC-MS/MS Chromatography and Mass Spectrometry Method

Analytes were resolved using an ultra-performance liquid chromatography system (Accela UPLC, Thermo Scientific, UK) equipped with a Biobasic AX 5 μm, 100 × 2.1 mm column (Thermo Electron Corporation, Murrieta, CA, USA) and a mobile phase consisting of a mixture of 10 mM NH_4_Ac in ACN/H_2_O (30:70 v/v), pH 6.0 (buffer A), and 1 mM NH_4_Ac in ACN/H_2_O (30:70 v/v), pH 10.5 (buffer B). The mobile phase gradient was employed, comprising: buffer A = 95% at 0–0.5 min, from 95 to 0% over 1.25 min, held at 0% for 1.75 min, from 0 to 95% over 0.1 min, ending with 95% for 2.9 min, all at a flow rate of 500 μl/min. Eluting compounds of interest were detected using a triple stage quadrupole Vantage mass spectrometry system (Thermo Scientific, UK) equipped with an electrospray ion source. Samples were analyzed by Multiple Reaction Monitoring, negative ion modes at a spray voltage of 3000 V. Nitrogen was used as sheath and auxiliary gas at a flow rate of 50 and 20 arbitrary units, respectively. Argon was used as collision gas with pressure of 1.5 mTorr. The optimum transitional daughter ions mass and collision energy (CE) of each analyst were as follows: ATP 506.0 → 134.1.2 (CE, 23 V), CTP 482.0 → 159.0 (CE, 35 V), dATP 490.0 → 159.0 (CE, 37 V), dCTP 466.0 → 159.0 (CE, 28 V), dTTP 481.0 → 159.0 (CE, 32 V), GTP 522.0 → 159.0 (CE, 43 V), UTP 483.0 → 159.0 (CE, 27 V) and ChloroATP 539.9 → 159.0 (CE, 43 V).

### Immunofluorescence

Cells were seeded on coverslips, fixed with 3.7% formaldehyde (15 min), permeabilized with 0.2% Triton-100 (5 min) and blocked in 3% BSA in PBS for 1 h at room temperature. Coverslips were incubated with primary antibody overnight at 4 °C, washed and incubated 1 h at room temperature with secondary antibodies and/or phalloidin conjugated to fluorophores. Coverslips were mounted with ProLong Diamond Antifade Mountant with DAPI (Molecular Probes) and imaged using a Zeiss 700 inverted microscope. Primary antibodies used were Flag M2 (#F3165) from Sigma; p34 (#07-227) from Millipore; Cortactin (#05-180) from Upstate. Alexa Fluor^®^ 488 phalloidin (#A12379) and other Alexa Fluor- conjugated secondary antibodies were from Invitrogen.

### Cell Spreading Assay

Cells were trypsinized, counted and seeded at 30,000 (HT1080) or 7,500 (MEFs) cells per well in 12-well plates pre-coated with fibronectin (5 μg/ml). Plates were incubated for 45 min before fixing with 3.7% formaldehyde (15 min), washing and staining with 0.1% Crystal Violet (30 min) at room temperature. Experiments were seeded in duplicate or triplicate and at least 5 fields of view were imaged per genotype. Cell area was manually quantified using ImageJ. For HT1080 cells, areas greater than 20,000 and less than 1000 were removed from the analysis. For primary MEF experiments, cell areas greater than 20,000 and less than 500 were removed from the analysis. Significance was analysed using one-way ANOVA plus Tukey’s multiple comparison’s test.

Single Cell Motility Assay. HT1080 cells were seeded at 6000 cells per well in ViewPlate-96 Black plates (Perkin Elmer# 6005182) in triplicate or quadruplicate. The next day, cells were labeled with 0.4 μM CellTracker™ Orange CMRA Dye (Invitrogen) for 30 min to allow better cell visualization. Cells were imaged on ImageXpress Micro XL Widefield High Content Screening System (Molecular Devices). 4 sites per well were imaged every 10 min for 80 time points. Motility analysis was performed using multidimensional motion analysis within MetaXpress software. Significance was analysed using one-way ANOVA plus Tukey’s multiple comparison’s test.

## Additional Information

**How to cite this article**: Moniz, L. S. *et al*. Phosphoproteomic comparison of *Pik3ca* and *Pten* signalling identifies the nucleotidase NT5C as a novel AKT substrate. *Sci. Rep.* **7**, 39985; doi: 10.1038/srep39985 (2017).

**Publisher's note:** Springer Nature remains neutral with regard to jurisdictional claims in published maps and institutional affiliations.

## Supplementary Material

Supplementary Figures

Supplementary Table S1

Supplementary Table S2

Supplementary Table S3

Supplementary Table S4

Supplementary Table S5

Supplementary Table S6

Supplementary Table S7

Supplementary Table S8

Supplementary Table S9
