# Supplementary material for: Phosphoproteomic comparison of Pik3ca and Pten signalling identifies the nucleotidase NT5C as a novel AKT substrate
Source: Sci Rep. 2017 Jan 6;7:39985. doi: 10.1038/srep39985 (PMC5216349; doi:10.1038/srep39985)
Supplement: Supplementary Figures [file srep39985-s1.pdf]

Phosphoproteomic comparison of *Pik3ca* and *Pten* signalling identifies the nucleotidase NT5C as a novel AKT substrate

Larissa S. Moniz[1], Silvia Surinova[1], Essam Ghazaly[2], Lorena Gonzalez Velasco[1], Syed Haider[2], JC Rodríguez-Prados[2], Inma M. Berenjeno[1], Claude Chelala[2] & Bart Vanhaesebroeck[1,]*

Supplementary Information

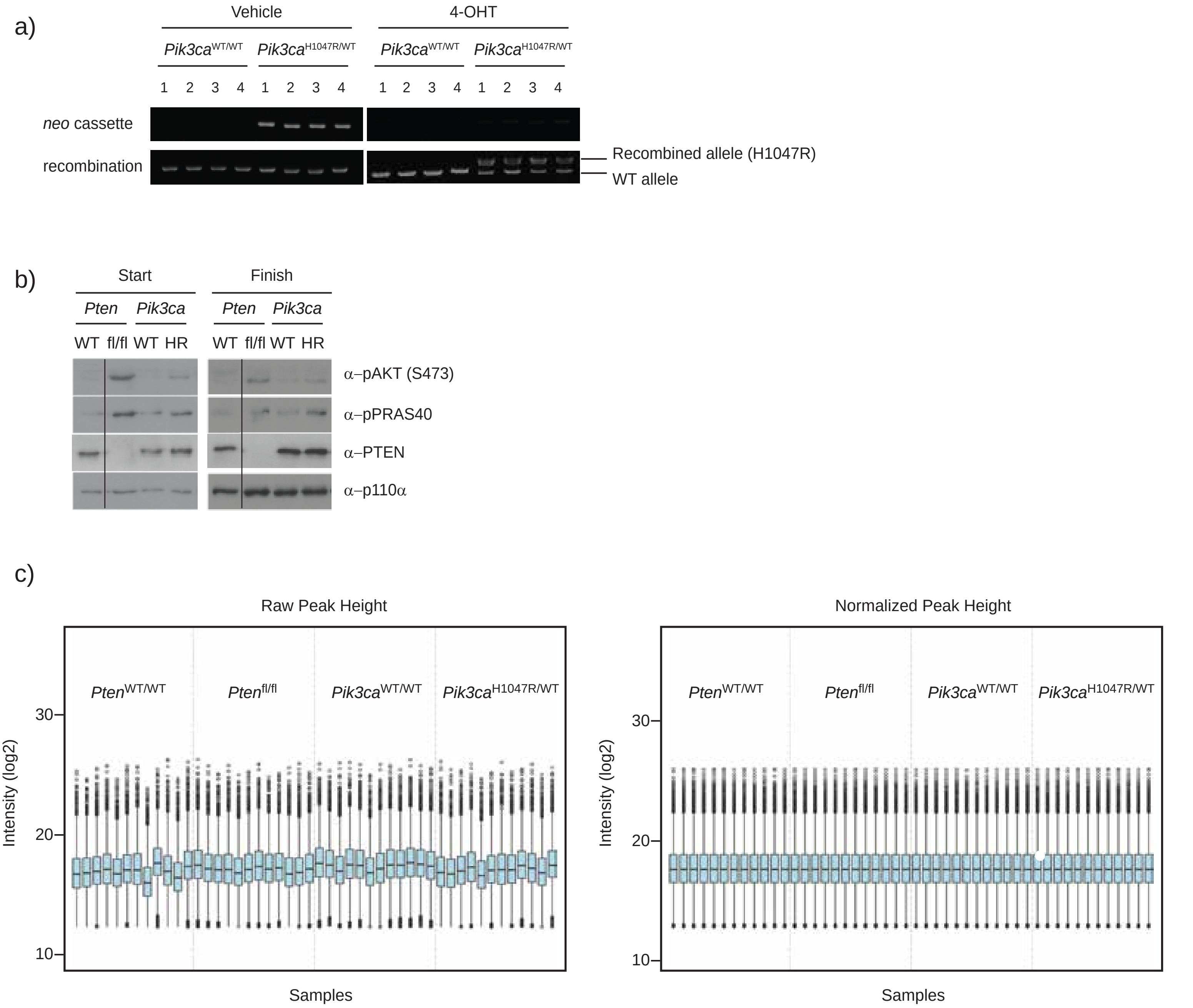

Supplementary Figure 1. Cellular models and phosphoproteomic workflow. (a) PCR analysis of representative samples from mass spectrometry screen. *Pik3ca*$^{H1047R/WT}$ or *Pik3ca*$^{WT/WT}$ MEFs were treated with 4-OHT or vehical 48h prior to DNA extraction. Each lane represents an independent MEF line. *Neo* cassette primers amplify a single 307-bp fragment that reveals the presence of the neo selection cassette. Recombination primers amplify a 340-bp fragment in the *Pik3ca*^{WT} allele (lower band) and a 425-bp fragment in the *Pik3ca*^{H1047R} allele (upper band). **(b)** Western blot controls from foci formation assay. Western blot analysis of 4-OHT-treated p53-immortalized MEFs gathered one day after seeding for foci formation (start) or at same time as foci formation assays were fixed (Finish). Images cropped from single gel, denoted by black line. **(c)** Total peak height intensities of each sample (*left*) before and (*right*) after normalization.

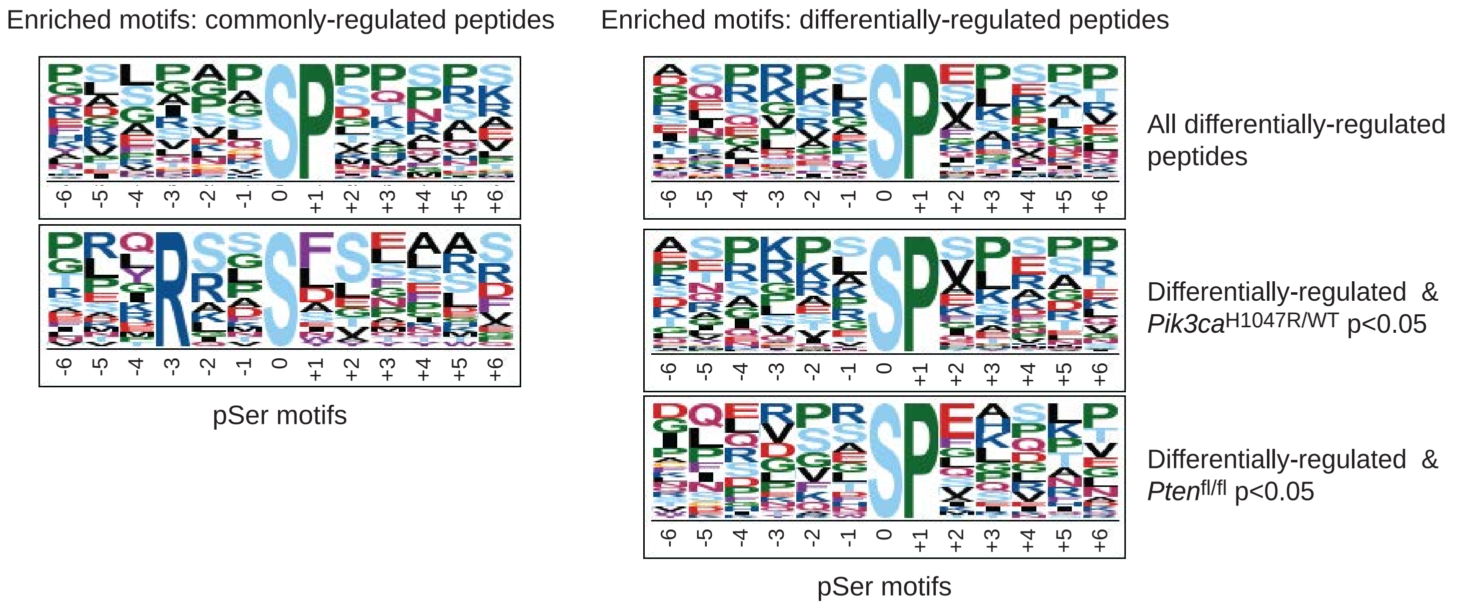

Supplementary Figure 2. Phosphoproteomic analysis of primary MEFs with inducible PI3K activation. Sequence logos of significantly enriched phospho-Serine (pSer) linear phosphorylation motifs identified using motif-x among (*left*) commonly-regulated and (*right*) differentially-regulated peptides.

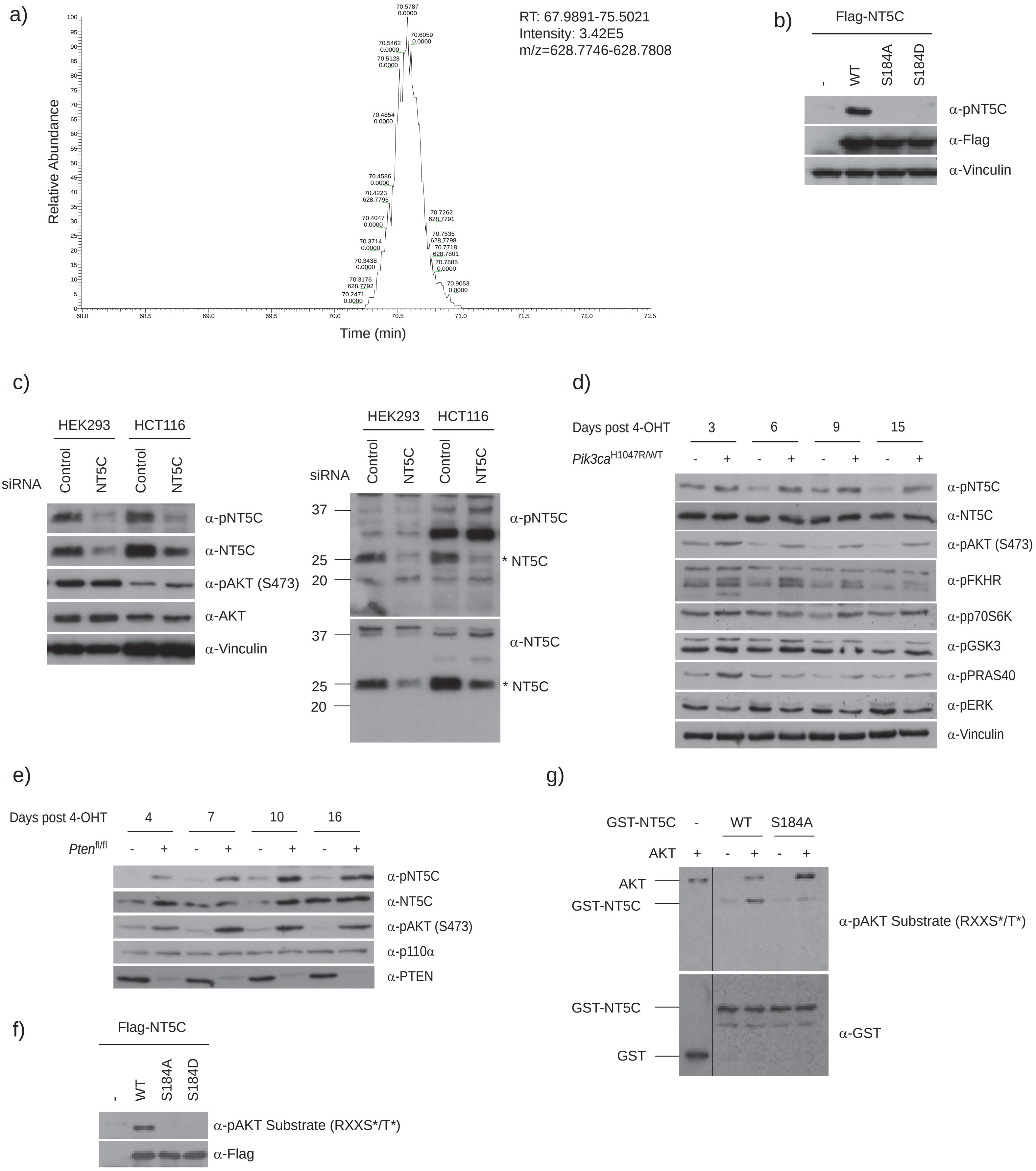

Supplementary Figure 3. Validation of NT5C as a novel AKT substrate. **(a)** MS/MS spectrum for NT5C peptide#5117 (p5117), LLS^WSDNWR, containing S184. RT (Retention time). **(b)** Validation of pNT5C antibody. Immunoblotting of lysates from primary MEFs stably expressing Flag-NT5C and indicated mutants. **(c)** Validation of pNT5C antibody. Immunoblotting of lysates from cells transfected with control of NT5C siRNA. (*left*) Cropped images. (*right*) Uncropped images. **(d,e)** Immunoblotting of lysates from primary MEFs at indicated days post 4-OHT treatment. **(d)** *Pik3ca*$^{WT/WT}$ cells denoted as (-);*Pik3ca*$^{H1047R/WT}$ cells denoted as (+) **(e)** *Pten*$^{WT/WT}$ cells denoted as (-);*Pten*$^{fl/fl}$ cells denoted as (+). **(f)** Validation of anti-AKT substrate antibody. Immunoblotting of lysates from HEK293 cells ectopically expressing NT5C and indicated mutants. **(g)** AKT *in vitro* kinase assay. Recombinant active AKT (200ng) was incubated with bacterially produced NT5C (1 μg). Phosphorylation was detected by immunoblotting with indicated antibodies. Images cropped from single gel, denoted by black line.

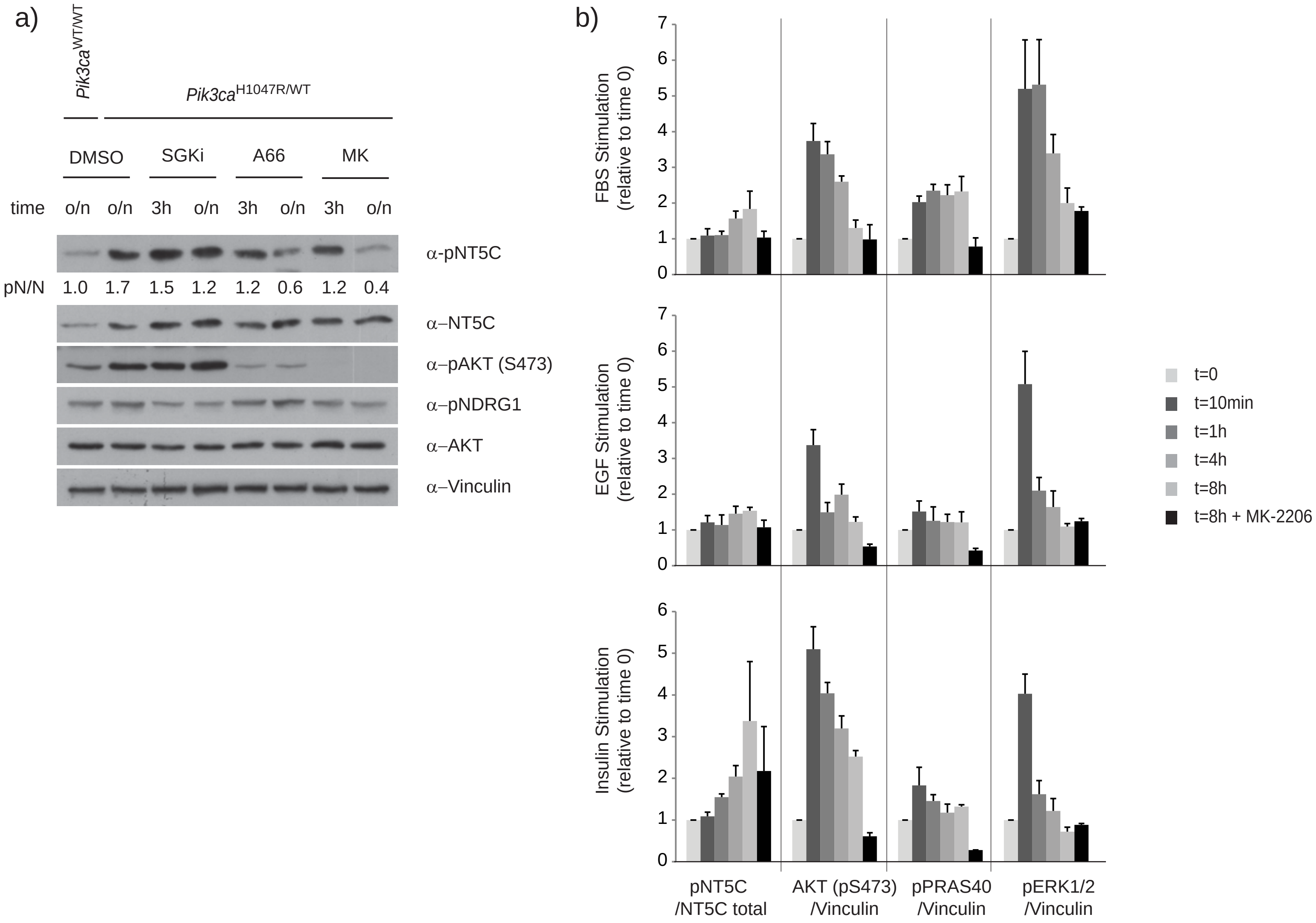

Supplementary Figure 4. Regulation of NT5C S184 phosphorylation. (**a**) Sensitivity of S184 phosphorylation to SGK1 inhibition. Primary MEFs were treated with A66 (3 µM), MK-2206 (MK) (1 µM), SGKi (GSK-650394) (10 µM) or DMSO as control for 3 h or overnight (o/n) before lysis. Lysates were immunoblotted as indicated. The ratio between pNT5C and NT5C total (pN/N) is indicated. (**b**) Comparison of phosphorylation kinetics in response to growth factor stimulation. Primary MEFs were starved overnight in 0.1% FBS and stimulated with FBS (10%), insulin (100 nM) or EGF (100 ng/ml) for the indicated times before lysis. DMSO or MK-2206 (1 µM) was added at same time as stimuli. Quantification of 3-5 independent experiments. Values are expressed relative to time 0 for each stimuli. Error bars are sem.

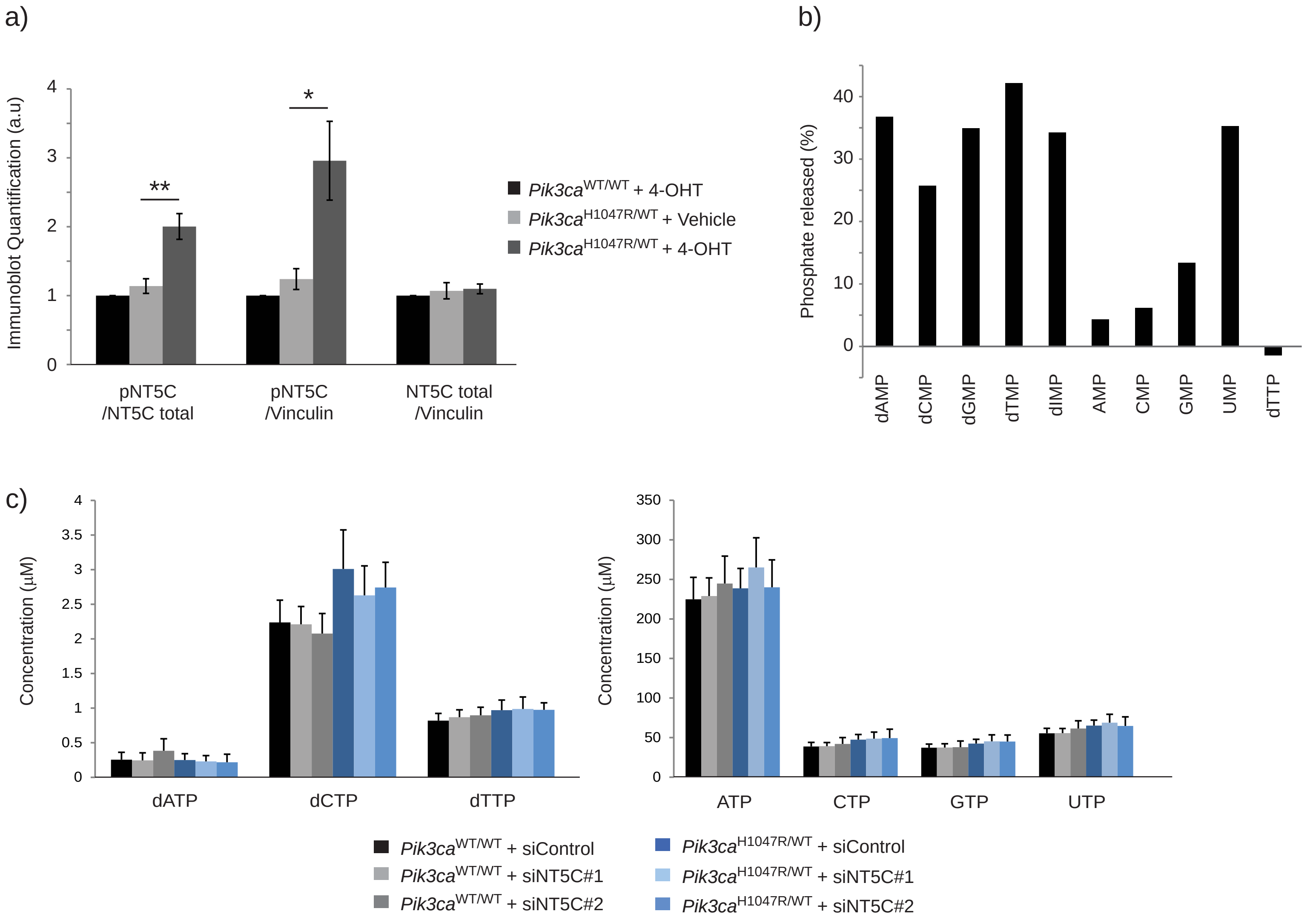

Supplementary Figure 5. Impact of S184 phosphorylation of NT5C catalytic activity. (**a**) S184 phosphorylation does not affect NT5C protein stability in primary MEFs. Quantification of Western blots from 4-5 independent experiments. Error bars are sem, *p<0.05, **p<0.01. (**b**) NT5C in vitro substrate specificity. Immunoprecipitates of Flag-NT5C ectopically expressed in HEK293 cells were incubated with 5 mM of the indicated nucleotides. Phosphate release was measured using a malachite green colorimetric assay and expressed as a percent of total nucleotide. (**c**) Effect of NT5C knockdown on cellular levels of (*left*) dNTP and (*right*) NTP. Nucleotides were extracted from primary MEFs, stably expressing indicated siRNA, and analysed by UPLC-MS/MS. Results are from 3 independent experiments. Error bars are sem.

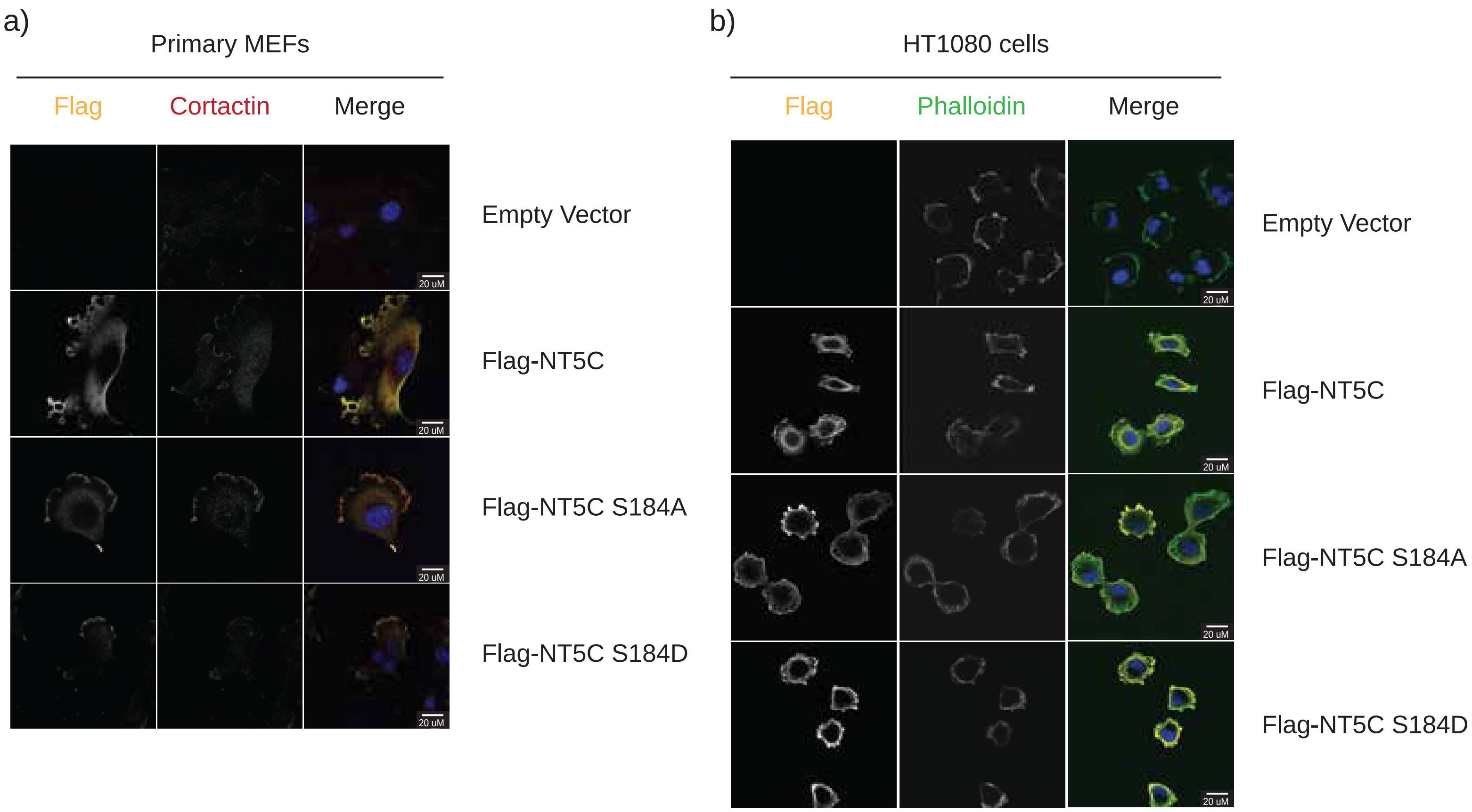

Supplementary Figure 6. NT5C interacts with the Arp2/3 complex. (**a**) NT5C localizes to cell edge and colocalizes with cortactin. Primary MEFs stably expressing Flag-NT5C constructs were fixed and stained with indicated antibodies. Cells were visualized by confocal microscopy (LSM Zeiss 700). Representative images are shown. (**b**) NT5C has limited localization with actin. HT1080 cells stably expressing Flag-NT5C constructs were fixed and stained with indicated antibodies. Fluorophore-conjugated phalloidin was used to visualize actin. Cells were visualized by confocal microscopy (LSM Zeiss 700). Representative images are shown.
